# Familial Cutaneomucosal Venous Malformations

**DOI:** 10.5826/dpc.1004a82

**Published:** 2020-10-26

**Authors:** Juan Francisco Mir-Bonafé, Marc Mir-Bonafé, Jaime Piquero-Casals, Eduardo Rozas-Muñoz

**Affiliations:** 1 Department of Dermatology, Hospital Son Llàtzer, Palma de Mallorca, Spain; 2 Department of Dermatology, Hospital Universitario Central de Asturias, Oviedo, Spain; 3 Department of Dermatology, Clínica Dermatológica Multidisciplinar Dermik, Barcelona, Spain; 4 Department of Dermatology, Hospital de San Pablo, Coquimbo, Chile

**Keywords:** venous malformations, TIE2, cutaneomucosal venous malformations

## Case Presentation

**Figure 1:**
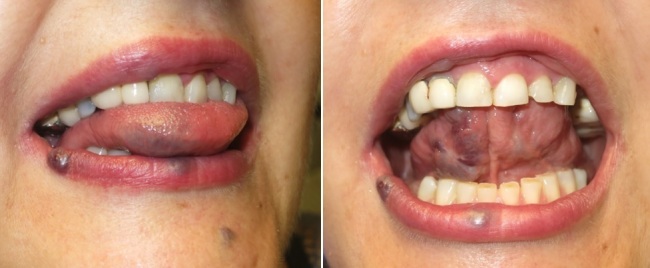
Multiple bluish soft nodules on the tongue, lips, oral mucosa, and perioral skin.

A Caucasian woman in her 40s, without relevant medical history, consulted the Dermatology Department for multiple bluish soft nodules on her tongue, lips, oral mucosa and perioral skin (Figure 1). The lesions had appeared during childhood and had grown progressively. She noted that her father had very similar lesions. An ultrasound showed venous malformation, and a diagnosis of familial cutaneomucosal venous malformations was made.

## Teaching Point

Familial cutaneomucosal venous malformations are the result of a germline mutation in the TIE2 receptor, with an autosomal dominant pattern of inheritance [1]. They usually present as multiple lesions that often appear from birth or during childhood and tend to grow throughout life. Even though they can be present in any location, they are more frequent on the face, with special predilection for the oral mucosa, lips and tongue. Rarely, they affect intestinal or anal mucosa. Venous malformations affecting the lips, tongue, or oral mucosa are rare. A diagnosis is based primarily on clinical findings. The differential diagnosis includes vascular tumors such as infantile hemangioma and pyogenic granuloma, venous lake, and other vascular malformations such as arteriovenous or lymphatic ones. The lips, tongue, buccal mucosa may be involved in another rare autosomal dominant disorder, Osler-Weber-Rendu syndrome, which is characterized by telangiectases resulting from arteriovenous malformations [2]. In doubtful cases, ultrasonography, MRI, a histopathological study, and genetic assays may be needed.
